# Possible involvement of the opioidergic system in the modulation of body temperature, jumping behavior and memory process in cholestatic and addicted mice

**DOI:** 10.17179/excli2019-2055

**Published:** 2020-03-04

**Authors:** Mohammad-Reza Zarrindast, Yasaman Issazadeh, Niloofar Rezaei, Fatemeh Khakpai

**Affiliations:** 1 Department of Pharmacology School of Medicine, Tehran University of Medical Sciences, Tehran, Iran; 2 Iranian National Center for Addiction Studies, Tehran University of Medical Sciences, Tehran, Iran; 3 Department of Neuroendocrinology, Endocrinology and Metabolism Clinical Sciences Institute, Tehran University of Medical Sciences, Tehran, Iran; 4 Cognitive and Neuroscience Research Center (CNRC), Tehran Medical Sciences, Islamic Azad University, Tehran, Iran

**Keywords:** cholestasis, withdrawal behaviors, morphine, tramadol, mice

## Abstract

Cholestasis is related to an increased plasma level of endogenous opioid levels. Naloxone-induced withdrawal syndrome has been reported in a mouse model of cholestasis. Moreover, studies revealed that the memory process is affected by cholestasis. Thus, we aimed at determining whether pharmacological manipulation of the opioidergic system is involved in signs of cholestasis disease such as hypothermia and withdrawal behaviors such as jumping behavior as well as memory process in mice. Cholestasis was induced by bile duct resection in mice and physical dependence was induced by administration of morphine and/or tramadol three times daily (8, 12 and 16 h) at the doses of 25, 50 and 75 mg/kg during three consecutive days. The memory process was assessed by a step-down passive avoidance test. Our results indicated that cholestatic mice showed hypothermia whereas cholestatic- and drug dependent mice indicated hyperthermia. Moreover, administration of morphine (50 mg/kg) and/or tramadol (50 mg/kg) on the 4th day, 2 h before naloxone injection significantly decreased latency to first jumping but increased the number of jumping and rearing behavior as well as locomotor activity in BDL-vs. sham-operated mice. In addition, the latency time of the step-down test decreased in BDL-vs. sham-operated group, showing impairment of memory in BDL mice. The results of this study support the evidence that (1) the opioidergic system involved in thermoregulation of cholestasis mice, (2) μ-opioid receptors play an important role in withdrawal behaviors, and (3) memory process is affected by cholestasis and addiction in mice.

## Introduction

Bile duct ligation (BDL) is a revealed animal model of cholestasis, with complete biliary obstruction and accumulation of many bile acids in liver and serum (Stedman et al., 2006[48]). BDL in mice incites typical time-dependent morphological and structural variations in the liver (Tag et al., 2015[49]). Patients with liver diseases and animal models of chronic liver failure may show hypothermia and remarkable impairment in cognitive functions such as memory process (Nasehi et al., 2013[37][40]). Researches have indicated that these behavioral presentations are associated with the altered neurotransmission and neural communication systems that involve opioids, dopamine, serotonin, noradrenaline, GABA, glutamate, and acetylcholine (Cauli et al., 2007[9]; Zarrindast et al., 2012[54]). Many studies demonstrated that endogenous opioid levels rise during cholestasis (Nelson et al., 2006[42]; Ahmadi et al., 2015[2]), and it is involved in the pathophysiology of cholestasis (Jones et al., 2002[23]; Bergasa, 2018[5]). 

The opioid system has three super-families of G-protein coupled receptors including µ, δ and κ of which, µ-receptor participates in main opioid actions, such as anti-nociception, anxiolytic, amnesic and thermoregulation effects (Rudy and Yaksh, 1977[47]; Zarrindast et al., 1995[55]; Zhu et al., 2011[57]; Nasehi et al., 2013[39]). Upon previous documents, there is enhanced opioidergic tone in cholestasis: (i) the opiate withdrawal-like response caused by an opiate antagonist in patients with cholestasis (Mansour-Ghanaei et al., 2006[33]; Hasanein et al., 2007[18]), (ii) the state of stereospecific, opiate antagonist reversible anti-nociception in animal model of cholestasis (Bergasa et al., 1994[6], 1997[7]), and (iii) the down-regulation of µ (and δ) opioid receptor in the brain membranes of rats with cholestasis (Bergasa et al., 1992[8]; Inan and Cowan, 2005[22]). 

Drug addiction is a type of chronic and relapsing disease of the brain (Leshner, 1997[30]). Continuous application of drugs, including opioids, leads to adaptive alterations in the CNS which are responsible for tolerance, physical dependence, and sensitization as well as relapse (Zarrindast et al., 2003[51]; Kotlinska and Bochenski, 2007[29]). The physical dependence on opioid drugs such as morphine and tramadol can be inferred from various aversive reactions and physiological signs following precipitation of withdrawal with narcotic antagonists for example naloxone. Between such signs, jumping is extensively considered the most sensitive and useful index of withdrawal intensity in rodents (particularly mice) and is the most usually used. Studies indicated that there is a positive relationship between the frequency of naloxone-precipitated withdrawal jumping and opioid agonist or antagonist dose (Kest et al., 2001[27], 2002[28]). 

Considering the role of opioidergic system in the pathophysiology of cholestasis (Jones et al., 2002[23]; Bergasa, 2018[5]) and the physical dependence as well as the role of this system in modulation of memory process, the main aim of this research is to report the effects of pharmacological manipulations of the opioidergic system in the modulation of body temperature, jumping behavior and memory process in cholestatic and addicted mice.

## Materials and Methods

### Animals

Male NMRI mice weighing 25-35 g (5-8 weeks old animals) were obtained from the Tehran University of Medical Sciences, Tehran, Iran. Mice were kept in groups of 10 in Plexiglas cages at constant room temperature (22 ± 2 °C) with a 12-h light/dark cycle (lights on 07:00 h) and relative humidity 45-55 %. Animals had free access to a standard balanced diet (Behparvar Co, Tehran, Iran, which comprised 22 % protein, 53 % carbohydrate, and 4.5 % lipid) and water ad libitum except during the limited periods of experiments. All experiments were performed during the light phase of the light/dark cycle. Each mouse handled previous to and during the course of the experiments for the purposes of gentling. Eight mice were used in each group and each mouse was used once only. In the present research, all experimental procedures and animal use were confirmed by the Research and Ethics Committee of the Faculty of Science; Tehran University of Medical Sciences.

### Bile duct ligation (BDL) surgery and induced cholestasis

There were two experimental groups: sham-operated (i.e. without BDL) and BDL mice. Laparotomy was done under general anesthesia induced by an intraperitoneal injection of ketamine hydrochloride (50 mg/kg) and xylazine (4 mg/kg). Sham-operation groups consisted of laparotomy and bile duct identification and manipulation without ligation or resection (with the purpose of measuring probable stress produced by surgery). In the BDL group, the main bile duct was first ligated using two ligatures about 0.5 cm apart and then transected at the midpoint among the two ligatures (Bergasa et al., 1994[6]). Sterile 0.9 % NaCl solution (1 ml/mice) injected intra-peritoneally instantly after surgery (to avoid the probable drop in the blood pressure due to the bloodshed in belly operation). All surgeries were done using an aseptic technique. In the instant after the operation, each mouse was placed in a cage by itself to avoid wound dehiscence and was moved to its original cage 4 h after surgery (Zarrindast et al., 2012[54]; Nasehi et al., 2013[39]). Operative mortality was less than 5 %.

### Induction of dependence 

5 days after BDL surgery, the mice were rendered dependent on morphine or tramadol, based on the method used previously (Zarrindast and Farzin, 1996[52]). Morphine sulfate was injected subcutaneously and tramadol was injected intra-peritoneally three times daily at 8, 12 and 16 h on the following dosage schedule. The three doses were 25, 50 and 75 mg/kg, respectively. The highest dose of the third daily administration was used to decrease any overnight withdrawal. Morphine and/or tramadol injection was carried out over a maximum of 3 days for any group of mice. A dose of 50 mg/kg of morphine sulfate or tramadol was also administrated on the 4^th^ day (2 h previous to naloxone injection). Loss of weight (8 - 9 %) and death (1 %) were observed with a chronic injection of morphine sulfate or tramadol.

### Temperature recording 

Body temperature was measured previous to surgery as a baseline temperature. Other than the baseline temperatures, temperatures were carefully recorded in zero (4 h after BDL surgery), three and six days after BDL surgery as well as 4^th ^day of drug dependence. For temperature measuring, the mice were located individually in experimental cages and body temperature was recorded three times in 10 min intervals. The mean was recorded in all experimental groups. This was to exclude the confounding influence of handling mice's temperature. After induction of addiction to morphine or tramadol in non-operated, sham-operated and BDL mice, body temperature was recorded through means of a rectal thermistor probe (Light Lab, Brighton, UK, sensitivity 0.1 °C). The probe was lubricated with petroleum jelly previous to being inserted within the rectum to a depth of 2 cm. The data are indicated as alterations in rectal temperature from the baseline values. Baseline values were those taken instantly previous to the BDL surgery. Experiments were carried out between 8 and 11 a.m.

### Naloxone-induced jumping 

Groups of 8 mice were tested for the occurrence of jumping after their tenth injection of morphine or tramadol on day 4 of drug dependence. Two hours after the last dose of morphine (50 mg/kg) or tramadol (50 mg/kg), abstinence was precipitated through subcutaneous injection of naloxone (2 mg/kg). Then the mice were located individually in a Perspex observation cylinder (15 cm diameter and 50 cm height). The number of jumps was measured instantly after naloxone administration over a 30 min period. 

### Memory apparatus and procedure

Mice were trained using a step-down passive avoidance task in accordance with previous researches (Nasehi et al., 2018[38]). The passive avoidance apparatus made of a wooden box (30 × 30 × 40 cm^3^) with a floor of parallel stainless steel rods (0.3 cm in diameter, spaced 1 cm apart). A wooden platform (4 × 4 × 4 cm^3^) was located at the center of the grid floor. Electric shock (1 Hz, 0.5 s, and 50 V DC) was given to the grid floor via an isolated stimulator (Grass S44; Grass Instruments, Quincy, Massachusetts, USA). During the training day, each mouse was slowly located on the wooden platform. When the mouse stepped down from the platform and located its all paws on the grid floor, it received foot shock for 15 s. Then, the mouse was immediately removed from the apparatus. On the test day, after 24 h, each mouse was located on the platform again and the step-down latency time was recorded by a stopwatch and considered as an index of passive avoidance test. If the mouse had not stepped down after a 5 min cut off time, the test was stopped. All behavioral tests were performed between 9 a.m. and 12 p.m. Table 1(Tab. 1) clarifies the protocol.

### Drugs 

The following drugs were used: morphine sulfate (Temad, Iran), Tramadol hydrochloride (Tehran Daru, Iran), and naloxone hydrochloride ampoules (Tolidaru, Iran). All drugs were dissolved in Sterile 0.9 % NaCl solution. Morphine and naloxone were injected subcutaneously but tramadol was injected intra-peritoneally in a volume of 10 ml/kg. The control groups received saline. The doses of drugs used were those active in previous researches (Zarrindast and Farzin, 1996[52]; Zarrindast et al., 1999[56], 2002[53]). 

### Experimental design

Three experiments have been designed. In experiment 1, body temperature was recorded previous to surgery, 4 h after BDL surgery, three and six days after BDL surgery as well as 4^th^ day of drug dependence. The mean was recorded in all experimental groups. In this experiment, 16 groups of animals were used. The eight sham-operated groups and eight BDL groups received saline (1 ml/kg, i.p.), naloxone (2 mg/kg), morphine (50 mg/kg), tramadol (50 mg/kg), morphine (50 mg/kg) plus tramadol (50 mg/kg), morphine (50 mg/kg) plus naloxone (2 mg/kg), tramadol (50 mg/kg) plus naloxone (2 mg/kg) and morphine (50 mg/kg) plus tramadol (50 mg/kg) plus naloxone (2 mg/kg). 

In experiment 2, naloxone-induced jumping behavior was measured. In this experiment, 6 groups of mice were used. The three sham-operated groups and three BDL groups received morphine (50 mg/kg) plus naloxone (2 mg/kg), tramadol (50 mg/kg) plus naloxone (2 mg/kg) and morphine (50 mg/kg) plus tramadol (50 mg/kg) plus naloxone (2 mg/kg). Latency to first jumping, numbers of jumping, rearing and locomotor activity were recorded for 30 min. In all experiments, the data from cholestatic mice were compared to respective sham-operated mice groups.

In experiment 3, memory process was measured after surgery and induction of drug dependence. In this experiment, 16 groups of animals were used. The 8 sham-operated groups and 8 BDL groups received saline (1 ml/kg, i.p.), naloxone (2 mg/kg), morphine (50 mg/kg), tramadol (50 mg/kg), morphine (50 mg/kg) + tramadol (50 mg/kg), morphine (50 mg/kg) + naloxone (2 mg/kg), and tramadol (50 mg/kg) + naloxone (2 mg/kg) as well as morphine (50 mg/kg) + tramadol (50 mg/kg) + naloxone (2 mg/kg). In this experiment, the data from cholestatic group were compared to respective sham-operated mice groups. Table 2(Tab. 2) explains the experimental groups.

### Statistical analysis

Two-way analysis of variance (two-way ANOVA) was used for the data comparison between groups. In the case of a significant F value, Post-hoc analysis (Tukey’s test) was done to evaluate specific group comparisons. A difference with P < 0.05 among the experimental groups was considered statistically significant. 

### Histology

**Figure 1:**
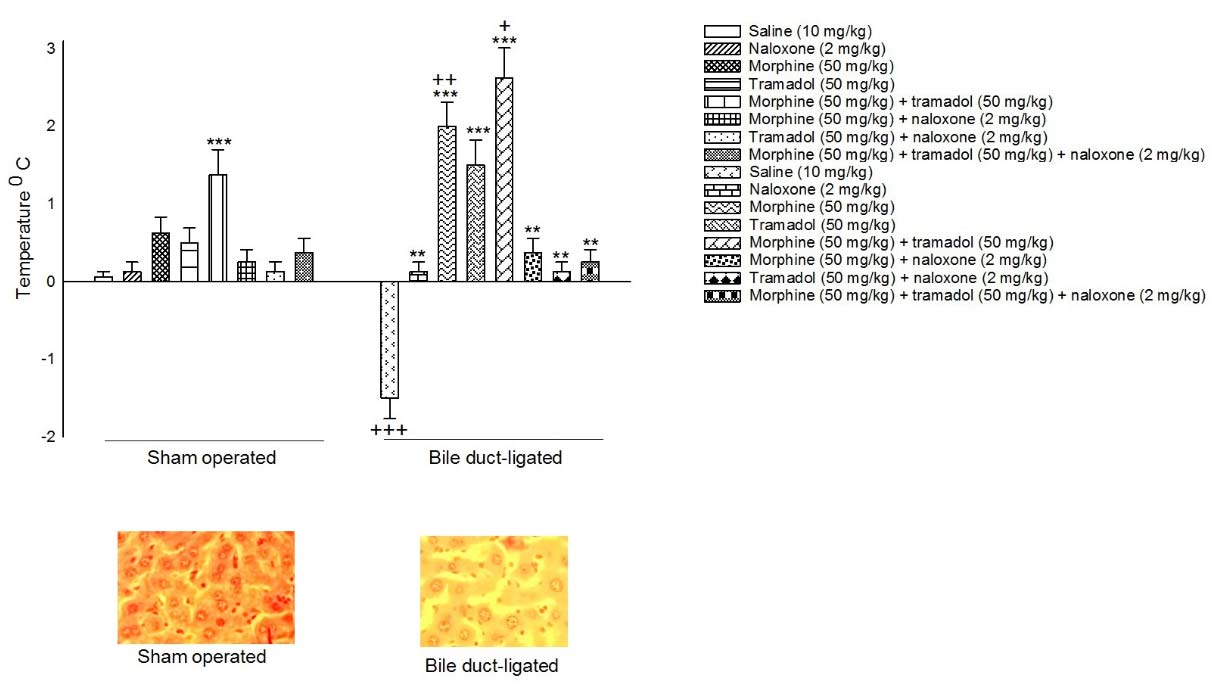
The effect of BDL and addiction to morphine and/or tramadol on body temperature. Sixteen groups of animals were used. The eight sham-operated groups and eight BDL groups received saline (10 mg/kg, i.p.), naloxone (2 mg/kg), morphine (50 mg/kg), tramadol (50 mg/kg), morphine (50 mg/kg) + tramadol (50 mg/kg), morphine (50 mg/kg) + naloxone (2 mg/kg), tramadol (50 mg/kg) + naloxone (2 mg/kg) and morphine (50 mg/kg) + tramadol (50 mg/kg) + naloxone (2 mg/kg). The animals' body temperatures were measured before surgery, 4 h after BDL surgery, three and six days after BDL surgery as well as 4^th^ day of drug dependence. The plots are expressed as mean ± S.E.M. **P < 0.01 and ***P < 0.001 when compared to the saline group and +*P *< 0.05, ++*P *< 0.01 and +++*P *< 0.001 when compared to the respective groups. Furthermore, Figure 1 shows histological sections of liver tissue in sham-operated and BDL mice. These histological sections differ for inter-cellular space and quantity of cells.

At the end of all experiments, liver tissues were inspected and processed to certify BDL surgery on tissue morphology. Liver tissues were fixed in neuter formol and buffered with 10 % phosphate and processed according to the routine method of paraffin inclusion. Histological sections of 4 µm were stained by the hematoxylin-eosin method for morphological evaluation (Figure 1(Fig. 1)) (Titford, 2005[50]; Llewellyn, 2009[32]).

## Results

### The effect of BDL and drug dependence on body temperature 

In Figure 1(Fig. 1), the effect of BDL and addiction to morphine and/or tramadol on body temperature is presented. As is shown in Figure 1(Fig. 1), BDL and addiction to morphine and/or tramadol changed body temperature [two-way ANOVA, BDL effect: F(1,112) = 5.342, P < 0.05; drug dependence effect: F(7,112) = 28.529, P < 0.001; BDL × drug dependence interaction: F(7,112) = 9.177, P < 0.001]. According to the Post-hoc analyses, BDL decreased but the injection of morphine, as well as morphine + tramadol, increased body temperature as compared to the sham-operated group. 

### The effect of BDL on the expression ofnaloxone-induced jumping behavior in drug dependent mice

**Figure 2:**
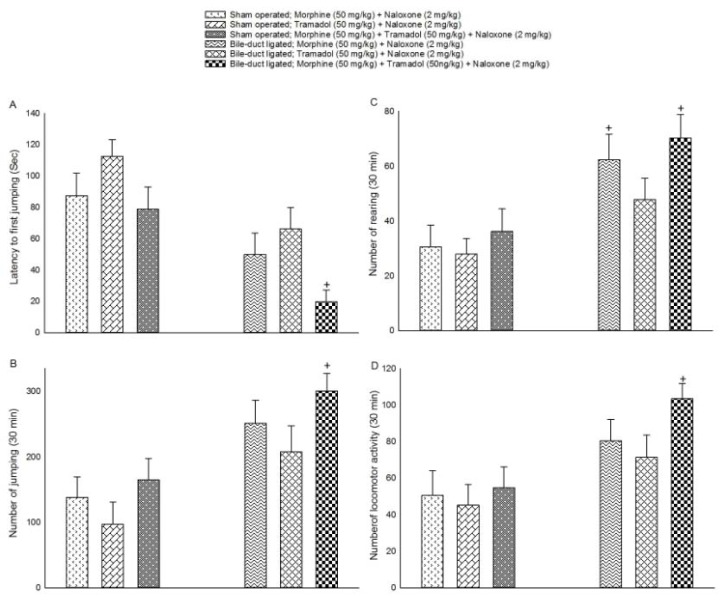
The effect of naloxone on withdrawal behaviors in cholestatic and addicted mice. Six groups of animals were used. The three sham-operated groups and three BDL groups received morphine (50 mg/kg) + naloxone (2 mg/kg), tramadol (50 mg/kg) + naloxone (2 mg/kg) and morphine (50 mg/kg) + tramadol (50 mg/kg) + naloxone (2 mg/kg). Latency to first jumping, numbers of jumping, rearing and locomotor activity were measured for 30 min. The plots are expressed as mean ± S.E.M. +*P *< 0.05 when compared to the respective groups.

The effect of BDL on the expression of naloxone-induced jumping behavior is shown in Figure 2(Fig. 2). The mice were divided into two groups: sham-operated and BDL groups. Both groups received morphine and/or tramadol (as described in 'Materials and Methods') to induce dependence as well as naloxone (2 h after morphine and/or tramadol injection). Two-way ANOVA followed by Tukey's test (shown in Figure 2A(Fig. 2)) revealed a significant effect of BDL [F (1,42)=21.395, P<0.001], and drug dependence [F (2,42)=5.082, P<0.05] but no significant effect of BDL × drug dependence interaction [F (2,42)=0.737, P>0.05] on latency to first jumping in BDL-vs. sham-operated animals. As shown in Figure 2B(Fig. 2) two-way ANOVA followed by Tukey's test indicated a significant effect of BDL [F (1,42)=19.172, P<0.001] but no significant effect of drug dependence [F (2,42)=2.884, P>0.05] and BDL × drug dependence interaction [F (2,42)=0.086, P>0.05] on jumping behavior in BDL-vs. sham-operated animals. In addition, two-way ANOVA followed by Tukey's test (shown in Figure 2C(Fig. 2)) exhibited a significant effect of BDL [F (1,42)=19.266, P<0.001] but no significant effect of drug dependence [F (2,42)=1.847, P>0.05] and BDL × drug dependence interaction [F (2,42)=0.448, P>0.05] on rearing behavior in BDL-vs. sham-operated animals. As presented in Figure 2D(Fig. 2) two-way ANOVA followed by Tukey's test displayed a significant effect of BDL [F (1,42)=13.806, P<0.001] but no significant effect of drug dependence [F (2,42)=1.688, P>0.05] and BDL × drug dependence interaction [F (2,42)=0.554, P>0.05] on locomotor activity in BDL-vs. sham-operated animals. Moreover, in this section, post-hoc analyses revealed that treatment with morphine + tramadol decreased latency to first jumping and increased jumping and rearing behavior as well as locomotor activity in BDL-vs. sham-operated animals.

### The effect of BDL and drug dependence on the memory process 

**Figure 3:**
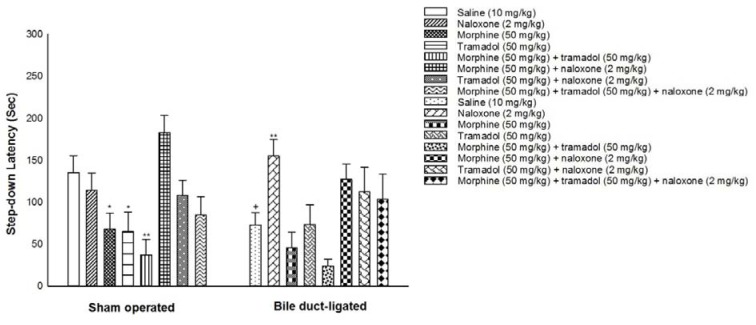
The effect of BDL and addiction to morphine and/or tramadol on memory process. Sixteen groups of animals were used. The eight sham-operated groups and eight BDL groups received saline (10 mg/kg, i.p.), naloxone (2 mg/kg), morphine (50 mg/kg), tramadol (50 mg/kg), morphine (50 mg/kg) + tramadol (50 mg/kg), morphine (50 mg/kg) + naloxone (2 mg/kg), tramadol (50 mg/kg) + naloxone (2 mg/kg) and morphine (50 mg/kg) + tramadol (50 mg/kg) + naloxone (2 mg/kg). Memory process was measured after surgery and induction of drug dependence. The plots are expressed as mean ± S.E.M. *P < 0.05 and **P < 0.01 when compared to the saline group and +*P *< 0.05 when compared to the respective group.

In Figure 3(Fig. 3), the effect of BDL and addiction to morphine and/or tramadol on the memory process is exhibited. As is shown in Figure 3(Fig. 3), two-way ANOVA followed by Tukey's test displayed no significant effect of BDL [F (1,112)=0.993, P>0.05] and BDL × drug dependence interaction [F (7,112) = 0.540, P>0.05] but a significant effect of drug dependence [F (7,112)=7.990, P<0.01] on memory process in BDL-vs. sham-operated animals. According to the post-hoc analyses, alone or co-administration of morphine (50 mg/kg) and tramadol (50 mg/kg) decreased memory in sham-operated mice while administration of naloxone (2 mg/kg) increased memory in BDL mice. In addition, the post-hoc analyses revealed that bile duct ligation impaired memory in BDL-vs. sham-operated mice.

For more results see the Supplementary data.

## Discussion

Cholestasis is a common pathological condition that can be induced in rodents by common BDL during surgical laparotomy (Georgiev et al., 2008[15]). It has been well-known that endogenous opioid levels enhance during cholestasis (Nelson et al., 2006[42]; Ahmadi et al., 2015[2]). This study explored the effect of BDL and drug dependence on the modulation of body temperature, jumping behavior and memory process in mice. First, the results revealed that cholestatic mice showed a significant decrease in body temperature whereas cholestatic- and drug dependent mice indicated a significant increase in body temperature. Second, naloxone (2 mg/kg) declined latency to first jumping but enhanced the number of jumping and rearing behavior as well as locomotor activity in BDL-vs. sham-operated mice, which may confirm an increase in endogenous opioids and activation of opioid receptors in this process. Finally, the memory process decreased two weeks after BDL in mice. 

In support of the present results, it has been reported that BDL declines body temperature (Moezi et al., 2006[35]; Nasehi et al., 2013[37]). Variation in the normal brain function is a property of both acute and chronic liver impairment which is recognized as hepatic encephalopathy. Hepatic encephalopathy is characterized by a deficiency in some neurotransmitter mechanisms in the brain (Zarrindast et al., 2012[54]). According to investigations, cholestasis caused hepatic encephalopathy would correspond to type C (Garcia-Moreno et al., 2005[14]), which is related to liver cirrhosis (Ferenci et al., 2002[12]). Neurological changes in these patients include variations in body temperature and cognition (Moezi et al., 2006[35]). Moreover, the effect of repeated exposure to morphine differs from the response system, dose, species, methodologies, and the ambient temperature which is being studied (Ganesan et al., 1991[13]; Gonzalez, 1993[16]; Nasehi et al., 2013[37]). In responses for example body temperature, morphine induces biphasic responses. Low doses of morphine (about 5 mg/kg) induce a hyperthermia effect that usually does not vary with repeated injections (Numan and Lal, 1981[43]; Baker and Tiffany, 1985[4]; Ganesan et al., 1991[13]). Higher doses of morphine (20-30 mg/kg) initially cause a hypothermic effect, while after repeated exposure, they cause a hyperthermia effect (Gunne, 1960[17]; Numan and Lal, 1981[43]; Ganesan et al., 1991[13]). There are documents explaining the opioidergic system variations seen in patients with liver diseases (Ebrahimkhani et al., 2006[11]). Endogenous opioids have been demonstrated to be enhanced during cholestasis and BDL (Ebrahimkhani et al., 2006[11]; Mombeini et al., 2006[36]). Several investigations have also revealed that several brain physiological and behavioral functions are affected by the opioidergic system (Ebrahimkhani et al., 2006[11]). Thus, in the current research, the cholestasis produced an increase in endogenous opioids may contribute to hypothermia but induction of dependence with repeated injections of morphine may cause hyperthermia. 

Investigations have usually reported that withdrawal of jumping behavior is the most reliable value for studying physical dependence in rodents, specifically with regard to opioids (Kest et al., 2001[27]; Cha et al., 2014[10]). Jumping response is described as an effort to escape from the test box during withdrawal (Liu et al., 1999[31]). Jumping expression in rodents is very variable and context-dependent (Azizi et al., 2012[3]). According to this study, alone administration of morphine or tramadol (for 3 days) and administration of naloxone on the fourth day did not change latency to first jumping, the number of jumping, rearing as well as locomotor activity in BDL-vs. sham-operated mice. On the other hand, injection of morphine along with tramadol (for 3 days) and administration of naloxone on the fourth day reduced the jumping latency period and increased the number of jumps, rearing, and locomotion in BDL-vs. sham-operated mice. We conclude that morphine and tramadol co-administration might have caused more intense escaping behaviors in mice. It seems that tramadol can enhance the depth of morphine dependence in mice, which may be due to the influence of tramadol on the opioid receptors (Raffa et al., 1992[45]).

In the next section of our study, we evaluated the effects of cholestasis and addiction on memory behavior. The obtained data indicated that the memory process decreased two weeks after BDL in mice. A growing body of researches reported that cholestasis affects brain functions (Jover et al., 2006[24]; Huang et al., 2009[21]). For example, animal studies have revealed an impaired object recognition memory (Garcia-Moreno et al., 2005[14]), spatial memory (Huang et al., 2010[20]), and working memory (Mendez et al., 2009[34]) in BDL rodents. Clinical studies have also indicated that patients with signs of hepatic encephalopathy may show a full-blown state of attention deficit as well as impairment of memory and cognitive function (Romero-Gomez et al., 2007[46]). The molecular signaling by which liver failure decreases cognitive function remains unknown. A number of investigations proposed that in the liver illness hyper-ammonia is one of the key factors responsible for the neurological variations (Pantiga et al., 2003[44]). Moreover, several documents indicated that some mechanisms for glutamatergic system participation in amnesia produced such as alteration of the brain nitric oxide, oxidative stress, disruption of calcium homeostasis, and membrane damage as well as cell death (Keller and Mattson, 1998[26]; Huang et al., 2010[20]; Nasehi et al., 2013[40]). All of the above mentioned biologic mechanisms can cause cognitive deficits in amnesia induced in BDL mice. In addition, we showed that alone or co-administration of morphine and tramadol in a single dose on the 4^th^ day impaired memory in sham-operated mice whereas administration of naloxone improved memory in BDL mice. We proposed that naloxone due to low dose could not affect memory but morphine and tramadol could impair memory through activation of the µ-opioid receptor in sham-operated mice. Also in BDL mice, the administration of naloxone improved memory in comparison with the saline group which was due to impairment of memory in cholestatic mice. Many investigators have documented that the chronic or acute use of opioid agonists like morphine and tramadol decreased memory process via the stimulation of µ-opioid receptor either in a direct or an indirect manner (Katzen-Perez et al., 2001[25]; Nava-Mesa et al., 2013[41]; Hosseini-Sharifabad et al., 2016[19]). Previous animal and clinical studies have revealed that opioidergic agents such as morphine and tramadol can modulate learning and memory behaviors, either in positive and negative directions (Nava-Mesa et al., 2013[41]; Hosseini-Sharifabad et al., 2016[19]). In agreement with this research, some studies indicated that the opioidergic system is involved in modulation of memory processes in cholestatic mice (Zarrindast et al., 2012[54]; Nasehi et al., 2013[40]; Afshari et al., 2018[1]). It can be concluded that the endogenous opioids involve in thermoregulation and withdrawal behaviors as well as the memory process of cholestasis mice. However, additional research is needed to clarify the exact mechanism of opioid receptors for inducing physical dependence in BDL mice as well as the nature of the progression of brain damage in cholestatic disease which caused memory deficit.

## Contributors

YI and NR acquired the animal data. FK wrote the manuscript. FK and MZ wereresponsible for the study concept, design, and assisted with the data analysis and interpretation of findings. All authors reviewed the content and approved the final version for publication.

## Acknowledgement

We are thankful to all contributors for their participation.

## Compliance with ethical standards

The study was carried out in accordance with ethical standards in all aspects.

## Conflict of interest

No financial or other conflicts of interest are declared.

## Supplementary Material

Supplementary data

## Figures and Tables

**Table 1:**
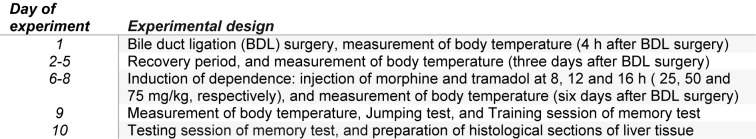
This table clarifies the experimental design.

**Table 2:**
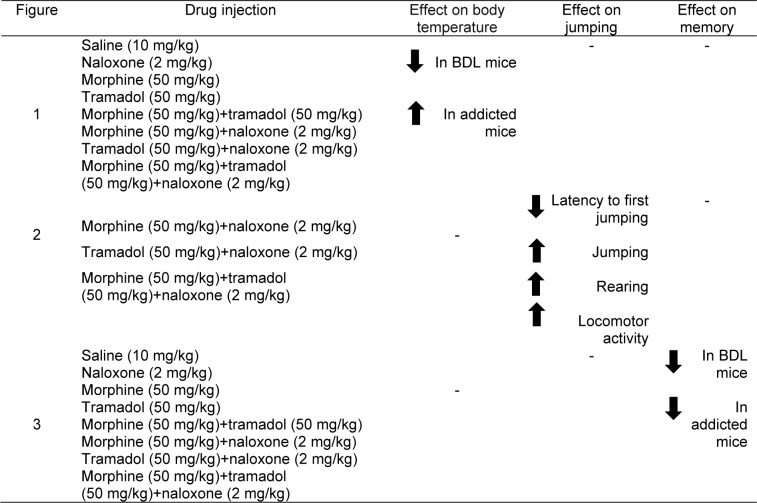
Table explains the experimental groups.
