# Supplementary material for: Possible involvement of the opioidergic system in the modulation of body temperature, jumping behavior and memory process in cholestatic and addicted mice
Source: EXCLI J. 2020 Mar 4;19:311–22. doi: 10.17179/excli2019-2055 (PMC7105937; doi:10.17179/excli2019-2055)
Supplement: Supplementary data [file EXCLI-19-311-s-001.pdf]

EXCLI Journal 2020;19:311-322 – ISSN 1611-2156
Received: December 11, 2019, accepted: February 24, 2020, published: March 04, 2020

Supplementary data to:

POSSIBLE INVOLVEMENT OF THE OPIOIDERGIC SYSTEM IN THE MODULATION OF BODY TEMPERATURE, JUMPING BEHAVIOR AND MEMORY PROCESS IN CHOLESTATIC AND ADDICTED MICE

Mohammad-Reza Zarrindast[a,b,c], Yasaman Issazadeh[a], Niloofar Rezaei[a], Fatemeh Khakpai[d,*]

[a] Department of Pharmacology School of Medicine, Tehran University of Medical Sciences, Tehran, Iran
[b] Iranian National Center for Addiction Studies, Tehran University of Medical Sciences, Tehran, Iran
[c] Department of Neuroendocrinology, Endocrinology and Metabolism Clinical Sciences Institute, Tehran University of Medical Sciences, Tehran, Iran
[d] Cognitive and Neuroscience Research Center (CNRC), Tehran Medical Sciences, Islamic Azad University, Tehran, Iran

* **Corresponding author:** Fatemeh Khakpai, Cognitive and Neuroscience Research Center (CNRC), Tehran Medical Sciences, Islamic Azad University, Tehran, Iran, P.O.Box: 193951495, Tel: +9821-22006660, Fax: +9821-22006660, E-mail: khakpai@iautmu.ac.ir

http://dx.doi.org/10.17179/excli2019-2055

This is an Open Access article distributed under the terms of the Creative Commons Attribution License (http://creativecommons.org/licenses/by/4.0/).

EXCLI Journal 2020;19:311-322 – ISSN 1611-2156
Received: December 11, 2019, accepted: February 24, 2020, published: March 04, 2020

Supplementary Table 1: The table indicates the data of body temperature in sham-operated and bile duct-ligated groups and refers to Figure 1 in the main text.

Sham-operated groups:

Saline (10 mg/kg)	Naloxone (2 mg/kg)	Morphine (50 mg/kg)	Tramadol (50 mg/kg)	Morphine (50 mg/kg)+ Tramadol (50 mg/kg)	Morphine (50 mg/kg) + Naloxone (2 mg/kg)	Tramadol (50 mg/kg) + Naloxone (2 mg/kg)	Morphine (50 mg/kg) + Tramadol (50 mg/kg) + Naloxone (2 mg/kg)
0.5	0	1	1	1	0	0	0
0	0	0	1	1	0	0	1
0	0	1	0	1	0	0	1
0	0	0.5	1	2	1	0	0
0	0	1.5	0	3	0	0	1
0	1	1	0	1	1	0	0
0	0	0	1	2	0	1	0
0	0	0	0	0	0	0	0

Bile duct-ligated groups:

Saline (10 mg/kg)	Naloxone (2 mg/kg)	Morphine (50 mg/kg)	Tramadol (50 mg/kg)	Morphine (50 mg/kg)+ Tramadol (50 mg/kg)	Morphine (50 mg/kg) + Naloxone (2 mg/kg)	Tramadol (50 mg/kg) + Naloxone (2 mg/kg)	Morphine (50 mg/kg) + Tramadol (50 mg/kg) + Naloxone (2 mg/kg)
-1	0	1	1	3	0	0	0
-1	0	2	1	1	1	0	0
-1	0	3	2	2	0	0	0
-1	0	3	0	2	1	0	0
-1	0	1	3	3	0	0	1
-2	0	3	2	3	0	0	0
-2	1	1.5	1	4.5	0	0	1
-3	0	1.5	2	2	1	1	0

EXCLI Journal 2020;19:311-322 – ISSN 1611-2156
Received: December 11, 2019, accepted: February 24, 2020, published: March 04, 2020

Supplementary Table 2: The table shows the data of latency to first jumping, number of jumping, rearing and locomotor activity in sham-operated and bile duct-ligated groups and refers to Figure 2 in the main text.

Latency to first jumping in sham-operated and bile duct-ligated groups (confers to Figure 2A).

Sham-operated groups			Bile duct-ligated groups		
Morphine (50 mg/kg) + Naloxone (2 mg/kg)	Tramadol (50 mg/kg) + Naloxone (2 mg/kg)	Morphine (50 mg/kg) + Tramadol (50 mg/kg) + Naloxone (2 mg/kg)	Morphine (50 mg/kg) + Naloxone (2 mg/kg)	Tramadol (50 mg/kg) + Naloxone (2 mg/kg)	Morphine (50 mg/kg) + Tramadol (50 mg/kg) + Naloxone (2 mg/kg)
60	120	90	20	0	0
100	180	60	0	50	0
100	100	20	50	120	8
180	100	130	50	80	20
60	120	50	92	40	50
64	80	100	100	100	40
75	100	50	8	90	40
60	100	130	80	50	0

Number of jumping in sham-operated and bile duct-ligated groups (confers to Figure 2B).

Sham-operated groups			Bile duct-ligated groups		
Morphine (50 mg/kg) + Naloxone (2 mg/kg)	Tramadol (50 mg/kg) + Naloxone (2 mg/kg)	Morphine (50 mg/kg) + Tramadol (50 mg/kg) + Naloxone (2 mg/kg)	Morphine (50 mg/kg) + Naloxone (2 mg/kg)	Tramadol (50 mg/kg) + Naloxone (2 mg/kg)	Morphine (50 mg/kg) + Tramadol (50 mg/kg) + Naloxone (2 mg/kg)
208	9	234	300	319	329
212	207	244	347	328	360
17	204	75	268	39	375
154	202	29	285	35	355
229	115	236	64	246	325
45	10	238	315	255	262
200	19	200	300	219	250
39	10	63	130	220	150

EXCLI Journal 2020;19:311-322 – ISSN 1611-2156
Received: December 11, 2019, accepted: February 24, 2020, published: March 04, 2020

Number of rearing in sham-operated and bile duct-ligated groups (confers to Figure 2C).

Sham-operated groups			Bile duct-ligated groups		
Morphine (50 mg/kg) + Naloxone (2 mg/kg)	Tramadol (50 mg/kg) + Naloxone (2 mg/kg)	Morphine (50 mg/kg) + Tramadol (50 mg/kg) + Naloxone (2 mg/kg)	Morphine (50 mg/kg) + Naloxone (2 mg/kg)	Tramadol (50 mg/kg) + Naloxone (2 mg/kg)	Morphine (50 mg/kg) + Tramadol (50 mg/kg) + Naloxone (2 mg/kg)
32	57	32	98	27	62
37	37	37	43	30	60
14	15	54	40	49	88
80	18	83	46	83	56
22	30	22	63	38	105
35	16	35	33	48	53
13	40	14	100	30	38
12	10	12	76	78	100

Number of locomotor activity in sham-operated and bile duct-ligated groups (confers to Figure 2D).

Sham-operated groups			Bile duct-ligated groups		
Morphine (50 mg/kg) + Naloxone (2 mg/kg)	Tramadol (50 mg/kg) + Naloxone (2 mg/kg)	Morphine (50 mg/kg) + Tramadol (50 mg/kg) + Naloxone (2 mg/kg)	Morphine (50 mg/kg) + Naloxone (2 mg/kg)	Tramadol (50 mg/kg) + Naloxone (2 mg/kg)	Morphine (50 mg/kg) + Tramadol (50 mg/kg) + Naloxone (2 mg/kg)
87	100	10	98	87	109
37	65	52	108	105	118
3	1	80	32	31	105
33	52	75	36	99	125
30	10	80	80	29	120
16	49	1	120	112	100
98	29	65	65	68	100
100	56	75	103	40	50

EXCLI Journal 2020;19:311-322 – ISSN 1611-2156
Received: December 11, 2019, accepted: February 24, 2020, published: March 04, 2020

Supplementary Table 3: The table explains the data of step-down latency in sham-operated and bile duct-ligated groups and refers to Figure 3 in the main text.

Sham operated groups:

Saline (10 mg/kg)	Naloxone (2 mg/kg)	Morphine (50 mg/kg)	Tramadol (50 mg/kg)	Morphine (50 mg/kg)+ Tramadol (50 mg/kg)	Morphine (50 mg/kg) + Naloxone (2 mg/kg)	Tramadol (50 mg/kg) + Naloxone (2 mg/kg)	Morphine (50 mg/kg) + Tramadol (50 mg/kg) + Naloxone (2 mg/kg)
250	50	80	8	100	250	76	5
145	70	23	196	10	200	73	35
143	105	13	130	20	163	74	28
150	238	160	2	15	140	32	172
90	120	107	29	136	98	125	167
100	150	8	40	5	174	160	100
60	80	56	60	2	166	171	96
145	100	98	55	10	273	154	75

Bile duct-ligated groups:

Saline (10 mg/kg)	Naloxone (2 mg/kg)	Morphine (50 mg/kg)	Tramadol (50 mg/kg)	Morphine (50 mg/kg)+ Tramadol (50 mg/kg)	Morphine (50 mg/kg) + Naloxone (2 mg/kg)	Tramadol (50 mg/kg) + Naloxone (2 mg/kg)	Morphine (50 mg/kg) + Tramadol (50 mg/kg) + Naloxone (2 mg/kg)
9	100	48	8	35	200	250	100
99	70	47	196	28	85	200	20
100	105	16	50	68	78	163	72
30	200	24	20	30	112	64	99
101	200	170	5	10	188	98	106
123	189	20	123	5	180	23	45
45	175	10	89	2	95	36	300
79	203	30	97	16	77	63	87
